# Eye behavior does not adapt to expected visual distraction during internally directed cognition

**DOI:** 10.1371/journal.pone.0204963

**Published:** 2018-09-28

**Authors:** Sonja Annerer-Walcher, Christof Körner, Mathias Benedek

**Affiliations:** Institute of Psychology, University of Graz, Graz, Austria; University of Wuerzburg, GERMANY

## Abstract

When focused on a specific internal task like calculating a multiplication in mind we are able to ignore sensory distraction. This may be achieved by effective perceptual decoupling during internally directed cognition. The present study investigated whether decoupling from external events during internally directed cognition represents an active shielding mechanism that adapts to expected external distraction or a passive/automatic shielding mechanism that is independent of external distraction. Participants performed multiplications in mind (e.g. 26 x 7), a task that required to turn attention inward as soon as the problem was encoded. At the beginning of a block of trials, participants were informed whether or not distractors could appear during the calculation period, thereby potentially allowing them to prepare for the distractors. We tracked their eye behavior as markers of perceptual decoupling and workload. Turning attention inward to calculate the multiplication elicited evidence of perceptual decoupling for five of six eye parameters: blink rate, saccade and microsaccade rate increased, gaze was less constricted to the center, and pupils dilated. Although participants perceived blocks with distractors as more challenging, performance and eye behavior markers of both perceptual decoupling and workload were unaffected. This result supports the notion of perceptual decoupling as an automatic mechanism: focusing inward induces desensitization to external events independent of external distraction.

## Introduction

Imagine thinking about a catchy first sentence for your manuscript while sitting in a park on a sunny summer afternoon. People walk by and are engaged in discussions, kids are playing with water balloons. The amount of irrelevant external distraction is immense. Yet, we seem to be very effective at ignoring everything around us when deeply focused on a mental task [1]. Several cognitive models (which we review next) assume that this is achieved by decoupling from sensory stimulation during internally directed cognition. It is still unclear whether perceptual decoupling represents an automatic mechanism that solely depends on internal task characteristics, or whether it actively adjusts to external distraction. The current study is the first to manipulate the expectation of visual distraction during an internal task to investigate whether perceptual decoupling prepares for visual distraction.

### Externally and internally directed cognition

Externally directed cognition (EDC) refers to cognition with main attentional focus on sensory information (e.g. reading the manuscript in the introductory example). Internally directed cognition (IDC), on the other hand, refers to cognition with main attentional focus on internal representations, such as memories and mental simulations (e.g. trying to come up with a good first sentence) which is largely independent of current sensory information [2]. Other terms used in the literature are outer and inner focus/attention [1], outward and inward attention/focus [1], externally and internally focused/directed cognition [2–5], internally- and externally-oriented processes [6] and stimulus-oriented and independent thought/cognition [7,8].

Results from dual-task paradigms show that EDC and IDC compete for common resources [9], and the competition increases with workload. For example, direct eye contact, a sensory stimulus with high social salience, is more disruptive to internal task performance than facing an averted gaze, especially when the demands of the internal task (e.g. verb generation) are high [10]. In a similar way, mind wandering during an external task (e.g. pressing a button every time a letter appears on screen except when the letter is a “C”, [11]) impairs performance in that task. Most tasks we perform in everyday life require a constant shift between EDC and IDC, for example, when we read this sentence (EDC) and then think about it (IDC).

Several models have been proposed that describe how IDC is maintained in face of external distraction. The *Perceptual Decoupling Hypothesis* [7] posits that an internal process (e.g. mind wandering) captures attentional resources, which entails a desensitization towards sensory stimulation [12]. This desensitization is evidenced by reduced cortical processing of sensory stimuli [12] and spontaneous eye behavior decoupled from sensory stimuli [13,14].

Largely consistent with the *Perceptual Decoupling Hypothesis*, the *Prefrontal-Basal Ganglia Working Memory Model* posits a basal-ganglia mediated gating process (for a review see [15]). The gate is open, when working memory is updated with useful information from the environment (EDC). “Otherwise, the gate is closed and irrelevant information is kept from needlessly occupying capacity.” [15]. The degree of gating by basal ganglia and other mechanisms like dopaminergic projections seems to depend on the demands on working memory updating [16] and individual differences in those systems [17,18].

It is less clear how perceptual decoupling depends on the level of visual distraction. In the present paper we focus on visual distraction that is largely predictable (i.e. one knows there will be a certain type of visual distractors, just not when). This is a common situation in everyday life, where irrelevant distraction is omnipresent and largely predictable but can vary substantially in its amount (e.g. sitting in the park and the number of people walking by varies). Yet, to our knowledge, there is no research directly investigating how expected visual distraction affects decoupling from external events.

### Eye behavior associated with IDC

As the need for sensory processing is reduced during IDC, eye behavior is less determined by the position and time of external stimuli–eye behavior decouples from external events [13]. The decoupling of eye behavior from external events can serve as a useful indicator of states of IDC. For example, reduced eye responses to visual stimuli in a choice reaction task were found to indicate mind wandering (e.g. [13]). It is less clear, however, how eye behavior responds to external stimulation during IDC, because during IDC visual stimuli represent distractors not targets.

Previous research suggest that eyes do not necessarily take a break and stay motionless during IDC. In fact, eye behavior can become coupled to events of imagined scenes. For example, when imagining a scene, one performs similar eye movements as one did during watching the scene in the first place [19,20]. These eye movements even occur, when eye movements are prohibited during encoding by maintaining central fixation, highlighting their possible functional role [19]. The stimuli-independent eye behavior elicited by internal processes can be used to infer perceptual decoupling without constantly probing perceptual decoupling through presentation of visual stimuli.

A variety of studies found eye behavior differing between EDC and IDC [3,13,21,22]. Here, we will consider six eye parameters that have been shown to be sensitive to the focus of attention: blink rate, saccade rate, microsaccade rate, fixation disparity, gaze position and pupil diameter.

People blink more often during IDC states like mind wandering or idea generation compared to reading [21,23]. They also blink more often right before they come up with a solution to a problem that requires insight [22].

Depending on the internal task and the external condition or task to which it is compared, the saccade rate can increase or decrease when turning attention inward. For example, solving anagrams in mind elicits fewer saccades than solving the anagram with the stimulus word still on the screen [3]. On the other hand, generating ideas in mind triggers more saccades than reading when only one letter at a time is presented [21]. The external task varied in gazing demands, with anagram solving requiring saccades to different letters whereas single-letter reading requires to focus on a fix position. In both studies, saccades during the internal condition were no longer coupled to the external stimulation but rather to internal processes (i.e. imagining ideas in mind).

In previous studies, microsaccade rate dropped when turning attention inward [3,21] and the fixation disparity increased (left and right eyes’ gaze position did no longer overlap; [3]). Both increased fixation disparity and decrease in microsaccades could mark decreased adaption of eye behavior to needs of visual processing (avoid blurring, double image and fading) due to dampening processing of sensory information on the cortical level.

Distraction may not just trigger passive decoupling of eye behavior from external events but maybe also active avoidance of distraction. For this reason, we also included gaze position as parameter. When solving a problem by insight, people look away from the area where the problem is presented more often than when solving it by an analytical approach [22]. Studies on gaze aversion [24,25] show that the active avoidance strategy of gaze aversion is sensitive to both saliency of visual distractors and cognitive load of the non-visual (internal) task. For example, gaze is more often averted during face-to-face questioning–which involves highly salient eye contact–than during video-mediated questioning [24]. Further, participants avert their gaze more often when the distractor becomes more salient (i.e., faster movement of the drifting grating in screen center, [25]). This effect did not appear when the main task was very easy; suggesting that adaption to distraction might only happen when the main task needs cognitive resources and does not allow for simultaneous processing of sensory information.

We additionally analyze pupil diameter as a marker of arousal due to workload [26–28]. Microsaccade rate is also affected by workload. Microsaccade rate increases when starting to perform a task (i.e. calculating multiplications in mind); however, this increase in microsaccade rate is less pronounced under higher workload [29].

### The current study

Most previous research has studied effects of visual distractors or mind wandering on performance of a task requiring EDC (e.g. visual search). In those paradigms, the external world has relevance for the main task and cannot be fully ignored. However, we also often engage in internal tasks for which the current visual environment is irrelevant or even disruptive. The current study is the first that manipulated expectation of visual distraction during an internal task to investigate whether perceptual decoupling prepares for visual distraction.

We designed a paradigm that allows us to investigate both (1) decoupling of eye behavior when turning attention inward and (2) the effect of expected distractor presence on this perceptual decoupling. The task (multiplications) first required encoding of external stimuli (reading the operands) followed by turning attention inward to solve the problem in mind (calculating the multiplication). Regarding the general effect of turning attention inward, we expected to see evidence of perceptual decoupling in terms of characteristic changes in various eye parameters. In this paradigm, the EDC phase (reading the operands) required hardly any eye movements because the external stimuli (operands) were presented in the center of the screen. We therefore expected eye behavior to be more active during calculating the multiplication in mind (IDC) than during reading the operands (EDC). Specifically, we expected more blinks, more saccades, more microsaccades, larger fixation disparity and less constriction of gaze to the center during IDC compared to EDC. As calculating multiplications increases workload, we expected pupils to dilate and microsaccade rate to increase.

To investigate the effect of expected distraction on perceptual decoupling, we designed a paradigm, in which changes in eye behavior due to (expected) distraction can be observed independently of changes to the display. We used a block design to strengthen the power of our analysis. In the no-distractor block, the multiplications were performed without distractors, whereas in the distractor block participants expected the occurrence of visual distractors. Numbers close to the result of the multiplications served as distractors. Processing of those numbers would impair calculation of the multiplications by consuming cognitive resources [9] and by interfering with the numbers manipulated in working memory. By analyzing periods without distractor presence from both blocks (i.e. initial 1.5 s of each trial), we were able to study whether eye behavior adjusted to the expected distractors by increasing perceptual decoupling or not. If perceptual decoupling adapts to expected distraction, we would expect stronger signs of perceptual decoupling in blocks with expected distractors than in those without. If perceptual decoupling is independent of expected distraction, decoupling of eye behavior should not differ between blocks with and without expected distraction.

We report how we determined our sample size, all data exclusions, all manipulations, and all measures in the study [30].

## Method

### Power analysis

To determine sample size, we performed a power analysis using G*Power (version 3.1.9; [31]). We planned and conducted a two-factorial repeated measures ANOVA with time and condition as independent variables. We set alpha-level to 0.05 and aimed to achieve a statistical power of 80% for a medium main effect (*d*_*z*_ = 0.50). This setting yielded 34 participants or more as required sample size. With the final sample of 36 participants, we achieved a power of 83% to detect a medium main effect (*d*_*z*_ = 0.50).

For the planned follow-up comparisons to investigate general perceptual decoupling (comparing reading the operands to calculating the multiplication) and the effect of distraction (comparing multiplications with and without distraction) we planned and conducted Bonferroni-corrected *t*-tests. Bonferroni-correction lowered alpha level to 0.017 and 0.013 resulting in a power of 69% and 65% to detect a medium sized difference (*d*_*z*_ = 0.50) with the final sample.

### Participants

Thirty-six adults (24 female, 12 male) aged 19 to 47 years (*M* = 25.75, *SD* = 6.02) participated in the experiment for payment (10 € per hour). Most participants were students (31 students). Twenty-three participants had normal vision and thirteen participants had corrected-to-normal vision (soft contact lenses, diopters range: -2.75–5.25) and reported no strabismus or other medical condition affecting vision. All participants gave written informed consent and the study was approved by the Ethics committee of the University of Graz. Three additional participants were excluded from analysis because they did not follow instructions in the passive viewing trials (they calculated the multiplications instead of passively view).

### Apparatus

The experiment took place in a sound-attenuated room with controlled illumination. Participants were seated in front of a 24 inch screen (1920 x 1080 pixels, 60 Hz refresh rate) at a distance of 70 cm, and their heads were stabilized by a chin rest.

Binocular eye data were recorded using an SMI RED250mobile system (RED: Remote Eye-tracking Device, SensoMotoric Instruments) with a temporal resolution of 250 Hz, a gaze position accuracy of 0.4° v.a., and a spatial resolution of 0.03°. The stimulus presentation was compiled in PsychoPy [32] using the Software Development Kit of SMI. There was a 9-point calibration procedure at the beginning of the two practice and main blocks and a drift check before each trial.

### Material

We chose multiplications as task for the current study as it requires both EDC and IDC: EDC when reading the operands and IDC when calculating the multiplication in mind (calculation period). The multiplication task allows us to compare EDC and IDC and to manipulate distractor presence within the calculation period. We used three conditions: multiplications without distraction, multiplication with distraction and passive viewing. Passive viewing served as control condition for effects in eye behavior that merely derived from changes in the visual display.

We compiled 44 different multiplications, with a two-digit first operand and a single-digit second operand (e.g. 24 x 8, see S3 Table).

We determined the type of multiplications best suited for our study by piloting three types of multiplications (ca. 10 problems per type) on lab members. Multiplications with two single-digit operands (e.g. 4 x 8) led to calculation times that were too short for distractor presentation. Multiplications with two two-digit operands (e.g. 24 x 18) led to too many errors and lack of motivation in participants. We chose multiplications with a two-digit and a single-digit operand (e.g. 24 x 8) because their calculation time was long enough for distractor presentation and they were doable enough to keep participants motivated.

Next, we generated the 44 multiplications (first operand two-digit, second operand single-digit) for the present study. Regarding the first operand (two-digit), we excluded double numbers (e.g. 22) and numbers ending with zero (e.g. 50). Regarding the second operand (single-digit), we chose numbers from 3 to 9, as we considered 0, 1 and 2 too easy. Then, we combined first and second operands to multiplication problems under the restriction that first operands (two-digit) can be used in two problems at max and each operands combination has to be unique. In the final sample of multiplications, all had a mental carry, except for three (two of which were in the passive viewing condition).

We divided the 44 multiplications into two sets of 22 multiplications each and randomly assigned 18 multiplications to actual multiplication and four to passive viewing (same for each participant). Order of sets and assignment to block with distractor and block without distractor were counter-balanced across participants. Order of trials within a set was randomized for each participant.

**Fig 1:**
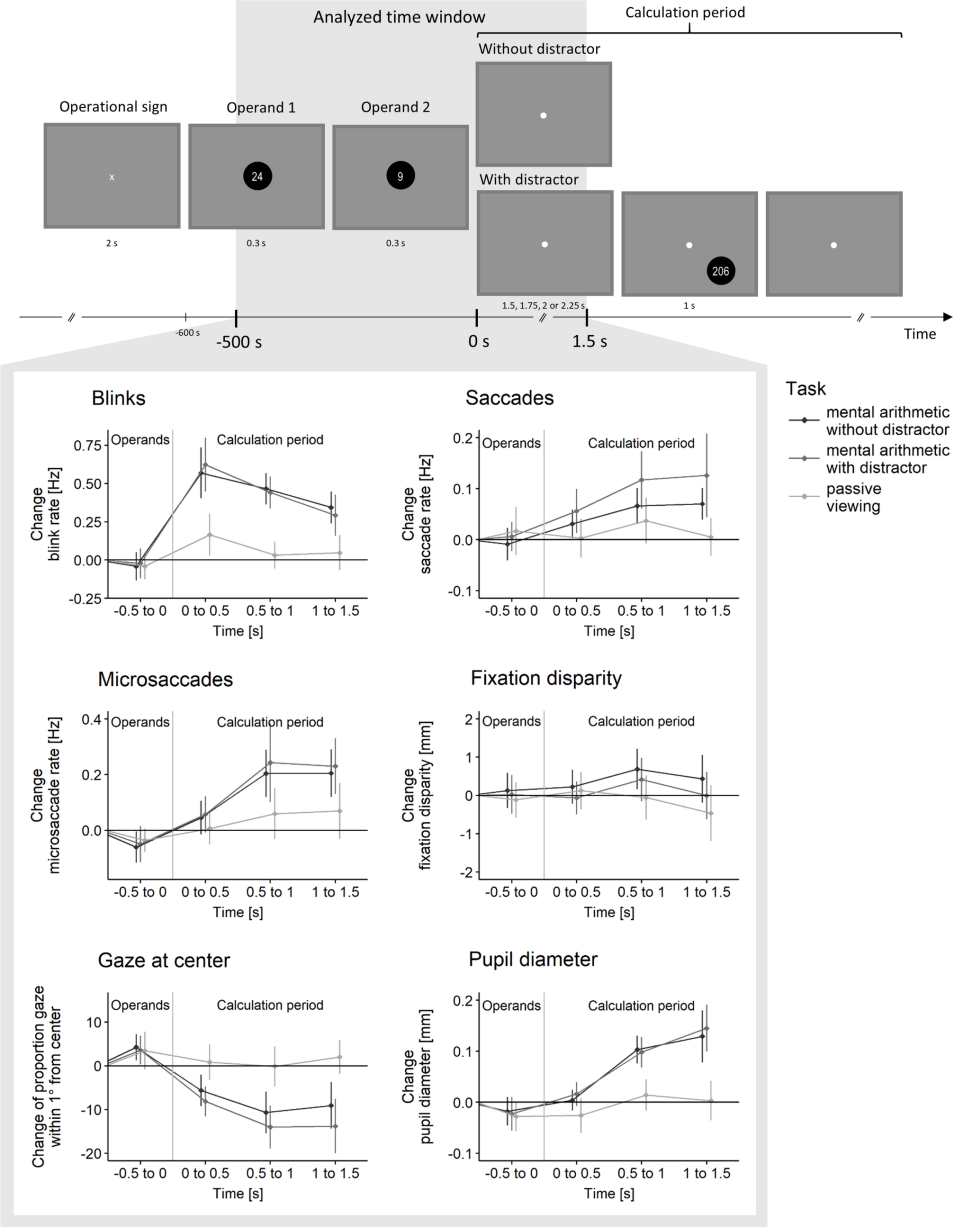
**Sequence of events in a trial (A) and results for six eye parameters (B) depending on time and condition.** Analyzed time window is highlighted grey. In trials which were not analyzed, the first distractor appeared already 1s after start of calculation period to guarantee that participants were expecting distractors even before the end of the 1.5s analysis window (catch trials). Eye parameters were baseline corrected. Error bars indicate 95% confidence intervals.

All stimuli were white and screen background was grey (see top of Fig 1). All numbers were presented inside a black circle of 70 pixels (ca. 1.57° v.a.) diameter at the screen center and of 80 pixels (ca. 1.79° v.a.) diameter on an imaginary circle (see below).

### Task and procedure

The sequence of events within a trial is visualized at the top of Fig 1 and was similar to the procedure in [29]. At the beginning of each trial, a short instruction told participants whether a multiplication trial (“Please calculate the multiplication”) or a passive viewing trial (“This is a baseline trial. Please just look at the screen center and press the mouse key when 5 seconds have elapsed”) followed. After participants pressed the spacebar and after a successful drift check, the multiplication sign (x, height: 27 pixels, ca. 0.6° visual angle) appeared at the center of the screen for 2 s. Then the first operand (two-digit) replaced the multiplication sign for 300 ms before it was itself replaced by the second operand (one digit) for another 300ms (both height: 27 pixels, ca. 0.6° v.a.). A small white circle (radius: 2.5 pixel, ca. 0.06° visual angle) appeared in the center of the screen and remained there for the rest of the calculation period. Participants were instructed to press the left mouse key as soon as they finished the calculation (multiplication trials) or as soon as they thought that 5 s had elapsed (passive viewing trials), respectively. Then, a response display was presented. It consisted of eight numerical response options to the multiplication (height 40 pixels, ca. 0.9° visual angle, the correct response and the distractors for each multiplication can be found in S3 Table) presented on an imaginary circle of 150 pixels in radius (ca. 2.24° visual angle) around the center of the screen. Participants selected an option via mouse click. We told participants to click in the middle of the circular response display (on the still present small white circle), if they had missed or forgotten the operands and therefore been unable to do the multiplication. We excluded those trials from analysis (see Table 1). In passive viewing trials, participants were required to click always in the middle of the response display. If they clicked anywhere else, the trial was excluded.

**Table 1:** Performance on the multiplication task and self-reports.

	Condition						
	Multiplication without distractor	Multiplication with distractor	*t*	*p*	Cohens’ *d*	*BF*_10_	*BF*_01_
Task performance:							
Correct Trials (%)	84.10 (13.62)	81.02 (12.05)	1.46	.154	0.24	0.47	2.12
Incorrect Trials (%)	8.80 (9.43)	10.96 (9.89)	1.27	.213	0.21	0.37	2.67
Forgot Trials (%)	7.10 (9.72)	8.03 (7.79)	0.68	.499	0.11	0.22	4.50
Median RT (s)	2.65 (0.77)	2.90 (1.38)	1.15	.259	0.19	0.33	3.05
Variation in RT (s)	2.69 (1.45)	2.88 (1.82)	0.60	.555	0.10	0.21	4.73
Self-report:							
Difficulty	2.71 (1.20)	3.37 (1.42)	4.46	< .001	0.74	287.12	0.00
Interestingness	3.49 (1.60)	3.77 (1.60)	2.25	.031	0.38	1.67	0.60
Effort	4.60 (1.52)	4.80 (1.41)	2.03	.051	0.34	1.11	0.90

Mean and Standard deviation in brackets. Self-reports were made on a scale ranging from 1 to 6. Critical *t*-value is 1.98, *df* = 35. BF10: Bayes Factor of alternative hypothesis compared to null-hypothesis, BF01: Bayes Factor of null-hypothesis compared to alternative hypothesis.

In the trials of the distractor block, a distracting number (that was close to the result of the multiplication) appeared after one of five possible intervals (1.0, 1.5, 1.75, 2, 2.25 s relative to the offset of the second operand). The 1s interval was included to guarantee that participants were expecting distractors even before the end of the 1.5s analysis window (see below). The 1s-interval trials were not analyzed. The distractor randomly appeared at one of eight positions on an imaginary circle (same circle as the one used for presentation of the response options). The distractor remained on screen for 500 ms.

Participants performed one block without distractors and one with distractors while we tracked their eye movements. Within each block, multiplication and passive viewing trials were randomized. Within the distractor block, each of the five possible distractor onset intervals were randomly assigned to four or five trials.

At the beginning of each block, we informed participants whether distractors would appear in this block or not and gave them three practice trials (two multiplication and one passive viewing trial). Half of the participants started with the block without distractor and half with the block with distractor. The two sets of multiplications were counterbalanced across distractor blocks and presentation order.

At the end of the experiment, participants answered three questions per distractor block regarding perceived difficulty (*1 = very easy* to 6 = very hard), interestingness (*1 = not at all* to *6 = very interesting*) and effort (*1 = not at all* to *6 = very much*) of the multiplications within this block. Additional short questionnaires regarding performance and current well-being were presented after each block. Those are not reported here but can be found in the open science framework (OSF, https://osf.io/nd475/). The present study was part of a 2.5 hour test session with other paradigms and questionnaires that are not related to the present paper. The present procedure took about 40 minutes and was administered at the beginning of that test session. The present procedure was followed by a second task irrelevant to the present study (i.e., eye tracking during a creativity task). Between tasks, participants filled out questionnaires and took breaks (2 breaks, at least 5 min per break).

### Data analysis

We performed data preprocessing and analysis in R [33]. We used the *ez* package [34] for ANOVAs, the *stats* package [33] for *t*-tests and the *BayesFactor* package [35] for calculating Bayes Factors (using the default setting *d* = .707).

#### Performance data

As measures of task performance in the multiplication trials, we calculated for each block (without and with distractor) percent correct trials, percent incorrect trials, percent forgotten trials, median response time (RT) and standard deviation of RT. RT was defined as time between disappearance of the second operand until the mouse click of participants. Percent incorrect trials served as primary measure of performance as it would best reflect successful interference of distracting numbers with the numbers held in mind.

#### Eye tracking data

From raw pupil and gaze position data, we excluded samples that occurred during a blink and additional 2 samples (= 8ms) at the beginning and end of a blink to remove distorted data due to lid closure (percentage data discarded: *M* = 6.87, *SD* = 5.53, *max* = 20.59). We also excluded samples with extreme values of pupil diameter or fixation disparity (pupil diameter: x > 15 mm or x < 1.8 mm; fixation disparity: x > mean inter-pupil distance of 60 mm) or three standard deviations beyond individual’s mean (percentage data discarded: *M* = 1.59, *SD* = 1.08, *max* = 4.50) to avoid data distortion due to measurement errors.

Saccades and microsaccades were determined using the Microsaccade Toolbox for R [36] with λ = 6 for velocity threshold and a minimum duration of 8 ms (two samples). Only binocular saccades with a minimum overlap of one sample were considered. We classified saccades smaller than or equal to 1.5° as microsaccades [37].

Pupil diameter and fixation disparity data were analyzed for time periods that were not classified as blink, saccade or microsaccade. Fixation disparity was calculated by subtracting the horizontal gaze position of the left eye from the right eye. This subtraction results in negative values when eyes are crossed (convergence, closer focus) and positive values when eyes are walled (divergence, farer focus). Microsaccades, blinks and saccades are presented in Hz, pupil diameter and fixation disparity are given in millimeter.

We analyzed correct trials only and discarded trials in which participants gave the wrong response to the multiplication or clicked in the middle of the panel indicating that they had forgotten the operands. Further, we excluded trials with RTs three standard deviations from participants mean RT (range 0–2 trials per participant, *M* = 1.05, *SD* = 0.60). Three participants had to be excluded from all analyses because they selected the correct solution to the multiplication instead of clicking in the middle of the panel in passive viewing trials indicating that they had ignored instructions of the passive viewing trials and always calculated the multiplications. All other participants responded correctly (clicked in the middle of the panel) in all passive viewing trials except for one participant, who responded incorrectly in two trials (leaving 6 trials).

There were no significant differences in any eye parameter in the passive viewing trials as a function of distractor presence. We therefore pooled passive viewing trials from the block without and from the block with distractors to increase power (8 instead of 4 trials). Note that the passive viewing trials served as a control condition for eye behavior changes elicited by changes in the visual display. The pooling of trials provided the opportunity to compare multiplication with and without distraction directly against passive viewing as an additional level of the variable condition (multiplications without distraction, multiplications with distraction, passive viewing).

We defined a time window starting at the presentation of the first operand and ending 1.5s after disappearance of the second operand. In this period, participants read the operands and performed the multiplication. We binned the eye-tracking data to four 500ms bins relative to start of the calculation period. There was one bin for the operands presentation and three bins for the calculation phase. Operands were presented for 600ms in total. To obtain equally sized time bins of 500ms, the time window for the operands started 100ms after their onset. Bin counts were calculated by summing up the number of saccade or blink onsets (and multiplying them with 2 to obtain saccade and blink rate per second), and calculating medians of pupil diameter and fixation disparity data. To measure if gaze was restricted to the center and avoided distractors, we defined a circular region of radius 1° around the center. In this region, only relevant information (operands) appeared but never distractors. Gaze at center was calculated by counting the number of samples in which gaze was within 1° from center and dividing it by the total number of samples per bin (500 ms = 125 samples, including blink periods) and multiplying the result with 100 to obtain percentages.

We were primarily interested in how eye behavior changes from the reading period to the calculation period as a function of expected distraction. We were not interested in differences in eye behavior that might have existed before stimulus onset (There were no such differences for any eye parameter as a function of condition (*F*(2,70) < 2.41, *p*s > .09, *η*^2^s < .035, *BF*_10_ < 0.42, *BF*_01_ > 2.42)). Therefore, we performed a trial-wise subtractive baseline correction on all bins. For pupil diameter and fixation disparity, we used the median of the 500 ms period preceding the start of the first operand as baseline. As saccades, blinks and microsaccades are relatively rare events (1–2 per second), a short window for assessing the baseline activity in one trial could lead to distortions. Therefore, we used mean rate per second from the whole 2 s presentation of the operational sign as baseline. To baseline-correct gaze at center, we also used the 2s window.

Magnitude of correlations between eye parameters ranged from .00 to .42 and are presented in S4 Table. The null, weak and moderate correlations indicate that our eye parameters were relatively largely from each other.

## Results

### Task performance and self-reports

Descriptive statistics of performance on the multiplication task and *t*-tests between distractor blocks are given in Table 1. To test whether distractor presence affected performance and/or self-reports in the multiplications, we calculated paired *t*-tests for each performance measure. Performance in the multiplications did not differ between task distractor blocks for any of the measures (see Table 1). However, participants rated multiplications with distractors as more difficult and more interesting than multiplications without distractors and tended to invest more effort.

### Eye behavior

Fig 1 illustrates changes of eye behavior from stimulus encoding (requiring EDC) to calculating the multiplication in mind (requiring IDC). To assess the development over time and the effect of expected distraction together, we performed two-factorial repeated-measures ANOVAs with time (-0.5s to 0s, 0s to 0.5s, 0.5s to 1.0s, and 1.0s to 1.5s) and condition (multiplication without distractor, multiplication with distractor, and passive viewing) as independent variables and baseline corrected eye behavior as dependent variable. Results of the repeated measures ANOVAs are presented in Table 2.

**Table 2:** Repeated measures ANOVAs for each eye parameter with condition (multiplication without distractor, multiplication with distractor, passive viewing) and Time (-0.5s to 0s, 0s to 0.5s, 0.5s to 1s, and 1s to 1.5s) as dependent variables.

Eye parameter	*F*	*p*	η^2^	*BF*_10_	*BF*_01_
*Blink rate*					
Condition	25.82	< .001	.097	11,900,000.00	0.00
Time	20.59	< .001	.160	3,330,000,000,000.00	0.00
Condition*Time	6.80	< .001	.039	2.39	0.42
*Saccade rate*					
Condition	3.91	.025	.026	4.35	0.23
Time	6.29	.001	.032	4.35	0.23
Condition*Time	2.66	.016	.017	0.06	17.33
*Microsaccade rate*					
Condition	3.39	.039	.024	3.13	0.32
Time	22.15	< .001	.112	115,000,000.00	0.00
Condition*Time	1.95	.074	.019	0.09	12.67
*Fixation disparity*					
Condition	1.51	.228	.012	0.28	3.52
Time	1.24	.299	.006	0.03	34.27
Condition*Time	1.02	.415	.006	0.01	81.22
*Gaze position*					
Condition	9.85	< .001	.072	53711.78	0.00
Time	15.96	< .001	.093	1,240,000.00	0.00
Condition*Time	5.69	< .001	.032	0.62	1.61
*Pupil diameter*					
Condition	10.12	< .001	.066	16222.21	0.00
Time	25.21	< .001	.147	192,000,000,000.00	0.00
Condition*Time	8.33	< .001	.038	1.81	0.55

Degrees of freedom: condition: 2, 70; time: 3, 105; condition*time: 6, 210. Bayes Factors refer to the main effects and interaction (not to likelihood of the whole model). BF10: Bayes Factor of alternative hypothesis compared to null-hypothesis, BF01: Bayes Factor of null-hypothesis compared to alternative hypothesis.

The main effect time, the main effect condition, and the interaction between time and condition were significant for most eye parameters including blink rate, saccade rate, proportion gaze at center, and pupil diameter. For microsaccade rate, there was a significant main effect of condition but no interaction. There was no effect on fixation disparity. The ANOVAs were followed up by two sets of planned comparisons, see below. The first set investigated the effect of turning attention inward for each condition and the second set investigated the effect of distractor expectation on the perceptual decoupling reaction within multiplication.

#### General effects of turning attention inward

First, we were interested whether eye behavior generally decoupled from external events after operands had been read and attention was turned inward to compute the multiplication. For each condition separately, we performed planned *t*-tests (Bonferroni corrected) comparing eye behavior between the period of operand presentation (-0.5s to 0s) and the period of calculation (three time bins from 0s to 1.5s). Means, Standard deviations and *t*-test statistic are presented in S1 Table; results are illustrated in Fig 1.

Calculating the multiplications in mind (both without and with distractor) increased blink rate (*t*(35)s > 3.83, *p*s < .002, *d*_*z*_s > 0.64, *BF*_10_s > 57.68) and saccade rate (without distractor: 0.5 to 1.5s, *t*(35)s > 2.77, *p*s < .026, *d*_*z*_s > 0.46, *BF*_10_s > 4.71; with distractor: 0.5 to 1s, *t*(35) = 2.96, *p* = .016, *d*_*z*_s = 0.49, *BF*_10_ = 7.14; see S1 Table and Fig 1). Further, microsaccade rate increased (*t*(35)s > 3.01, *p*s < .014, *d*_*z*_s > 0.50, *BF*_10_s > 7.91), proportion of gaze at center decreased (*t*(35)s > 3.61, *p*s < .001, *d*_*z*_s > 0.66, *BF*_10_s > 80.48) and pupil diameter increased (without distractor: 0.5 to 1.5s, *t*(35)s > 4.50, *p*s < .001, *d*_*z*_s > 0.75, *BF*_10_s > 337.73; with distractor: 0 to 1.5s, *t*(35)s > 3.53, *p*s < .003, *d*_*z*_s > 0.59, *BF*_10_s > 27.47) when calculating the multiplication. There was no change in fixation disparity (*t*(35)s < 1.56, *p*s > .380, *d*_*z*_s < 0.26, *BF*_10_s < 0.55). Taken together, five out of six eye parameters showed significant changes from stimulus encoding (EDC) to calculating the multiplications (IDC, see Fig 1). In the passive viewing trials, eye behavior did not differ between operands presentation and calculation period (*t*(35)s < 2.27, *p*s > .088, *d*_*z*_s < 0.38, *BF*_10_s < 2.42), except for a release of blinks right after operand disappearance (*t*(35) = 2.64, *p* = .036, *d*_*z*_s = 0.44, *BF*_10_ = 3.62, see Fig 1 and Table 2).

For the sake of completeness, we also compared passive viewing to the two multiplication conditions (see S1 Table for means and standard deviations and S2 Table for *t*-test statistics). During operand presentation (-0.5 to 0s), passive viewing did not differ from the two multiplication conditions (*t*(35)s < 1.47, *p*s > .600, *d*_*z*_s < 0.25, *BF*_10_s < 0.47). Within the calculation period, blink rate, gaze position and pupil diameter differed significantly between the passive viewing condition and the two multiplication conditions (from 0.5s to 1.5s: *t*(35)s > 2.82, *p*s < .031, *d*_*z*_s > 0.47, *BF*_10_s > 5.22), further suggesting that eyes decoupled in the multiplication conditions but not in the passive viewing condition. Saccade rate, microsaccade rate and fixation disparity were similar during passive viewing compared to the two multiplication conditions (*t*(35)s < 2.44, *p*s > .080, *d*_*z*_s < 0.41, *BF*_10_s < 2.37).

#### Effect of expected distraction

In a next step, we investigated whether perceptual decoupling in the multiplication trials increases when expecting distraction by comparing multiplications without and with distractor with planned *t*-tests (Mean and standard deviations are given in S1 Table, *t*-test statistics are given in Table 3). Eye behavior during calculating the multiplication did not differ as a function of distractor presence (with or without distractors) for any eye parameter at any time point (*t*(35)s < 2.07, *p*s > .186, *d*_*z*_s < 0.35, *BF*_10_s < 0.71; see Table 3 and Fig 1). These results suggest that perceptual decoupling does not adapt to expected visual distraction.

**Table 3:** Planned comparisons of eye behavior during multiplications between without vs. with distractor.

Eye parameter	Time	Without distractor	With distractor	*t*	*p*^a^	Cohen’s *d*	*BF*_10_	*BF*_01_
Blink rate	-0.5 to 0s	-0.04 (0.27)	-0.02 (0.29)	0.58	1	0.10	0.21	4.77
	0s to 0.5s	0.57 (0.49)	0.62 (0.52)	0.78	1	0.13	0.24	4.20
	0.5s to 1s	0.47 (0.3)	0.44 (0.31)	-0.38	1	-0.06	0.19	5.23
	1s to 1.5s	0.34 (0.31)	0.29 (0.4)	-0.74	1	-0.12	0.23	4.33
Saccade rate	-0.5 to 0s	-0.01 (0.1)	0.01 (0.09)	2.06	.187	0.34	1.18	0.85
	0s to 0.5s	0.03 (0.08)	0.06 (0.13)	1.47	.606	0.24	0.48	2.10
	0.5s to 1s	0.07 (0.1)	0.12 (0.17)	1.75	.355	0.29	0.71	1.41
	1s to 1.5s	0.07 (0.09)	0.13 (0.24)	1.37	.721	0.23	0.42	2.38
Micro-saccade rate	-0.5 to 0s	-0.06 (0.17)	-0.05 (0.19)	0.31	1	0.05	0.19	5.35
	0s to 0.5s	0.05 (0.18)	0.06 (0.19)	0.27	1	0.05	0.19	5.40
	0.5s to 1s	0.2 (0.25)	0.24 (0.42)	0.57	1	0.09	0.21	4.81
	1s to 1.5s	0.21 (0.25)	0.23 (0.3)	0.35	1	0.06	0.19	5.27
Fixation disparity	-0.5 to 0s	0.13 (1.35)	0.02 (1.49)	-0.35	1	-0.06	0.19	5.28
	0s to 0.5s	0.23 (1.31)	-0.06 (1.26)	-0.98	1	-0.16	0.28	3.59
	0.5s to 1s	0.69 (1.55)	0.41 (1.67)	-0.82	1	-0.14	0.24	4.08
	1s to 1.5s	0.43 (1.84)	0 (1.82)	-1.05	1	-0.17	0.30	3.38
Gaze position	-0.5 to 0s	4.24 (8.85)	3.56 (9.64)	-0.48	1	-0.08	0.20	5.03
	0s to 0.5s	-5.64 (10.57)	-8.15 (10.14)	-1.23	.914	-0.20	0.36	2.80
	0.5s to 1s	-10.69 (14.08)	-14 (14.49)	-1.22	.917	-0.20	0.36	2.81
	1s to 1.5s	-9.09 (15.74)	-13.81 (18.43)	-1.73	.373	-0.29	0.68	1.46
Pupil diameter	-0.5 to 0s	-0.02 (0.08)	-0.02 (0.1)	-0.51	1	-0.08	0.20	4.96
	0s to 0.5s	0 (0.06)	0.02 (0.07)	0.76	1	0.13	0.23	4.28
	0.5s to 1s	0.1 (0.08)	0.1 (0.09)	-0.24	1	-0.04	0.18	5.44
	1s to 1.5s	0.13 (0.15)	0.15 (0.14)	0.85	1	0.14	0.25	4.01

To analyze the effect of distractor presence on the strength of perceptual decoupling, we compared the two conditions multiplication without and with distractor for each time bin. Mean and standard deviation in brackets are given for each condition and time bin. BF10: Bayes Factor of alternative hypothesis compared to null-hypothesis, BF01: Bayes Factor of null-hypothesis compared to alternative hypothesis.

^a^ *p*-values were corrected for 4 tests per eye parameter using Bonferroni-correction.

*df* = 35. Significant *t*-values are highlighted in bold.

## Discussion

Turning attention inward to calculate multiplications in mind led to a characteristic response in five out of six eye parameters. People blinked more often, made more saccades and microsaccades, restricted their gaze less to the center, and their pupils dilated. Whether distractors were to be expected or not during the calculation of multiplications had no effect on this perceptual decoupling of eye behavior. In the following, we discuss these findings in the context of available models of IDC and EDC.

### Decoupling

The current study found further support for the *Perceptual Decoupling Hypothesis* [7]. Turning attention from external stimulation (the operands on the screen) inward (solving the multiplication in mind) led to a strong decoupling of eye behavior from external stimulation. While reading the operands required little eye movements, calculating the multiplication in mind increased spontaneous eye activity. Such an effect did not emerge for the passive viewing trials, underlining that the effect did not result from visual changes due to disappearance of the operands.

This stimulus-independent increase in eye activity is in line with results from mind wandering and creativity research. For example, pupils show increased spontaneous activity during mind wandering [13] and blinks and eye movements occur more often during idea generation than during reading one letter at a time [21]. As focused search for sensory information is not required by internal tasks, eye behavior decouples from sensory information [3,21], which allows for increased spontaneous eye activity. Additionally, the spontaneous eye activity could reflect potential manifestations of internal processes [19,20] (e.g. visualization of the multiplication in mind).

### Effect of distractor presence

Participants perceived the distractor block as more challenging and interesting than the block without distraction. However, whether a distractor was expected or not did not influence performance nor did it influence how strongly the eyes decoupled. This result indicates that during IDC, perceptual decoupling seems not to adapt to expected visual distraction. Some participants actually reported that they had not noticed any distractors during all multiplication trials, further underlining the strength of perceptual decoupling during demanding internal tasks.

Please note here that although our data yielded no or only anecdotal support (*BF*_10_s < 1.17) for the hypothesis that eye behavior adapts to expected distraction, support for the null hypothesis was substantial at best but not strong (*BF*_01_s < 5.35) [38]. One reason for that may be the number of trials per condition (18) which was held rather low to avoid exhaustion and demotivation. Therefore, our results require replication in future studies with a larger number of trials and/or participants.

Regarding performance measures, research on dual-task paradigms (both EDC) showed that the mere anticipation of a cognitively demanding secondary task impairs performance on the first task [39,40]. If we consider inhibition of interfering distractors in our paradigm as a secondary task, the mere expectation of distractors should have affected performance on the multiplication trials in form of more errors and/or prolonged RTs compared to the no-distractor condition. Lack of such an effect suggests that perceptual decoupling is rather an automatic process associated with IDC than an active inhibition of secondary-task stimuli.

Results from gaze aversion research may have suggested some adaptation of eye behavior to the distractor condition [24,25,41]. Relevant research showed that distracting stimuli are avoided more often when saliency of visual distractors and cognitive load of the internal task are high. In those studies, distractors had very high saliency, such as eye contact [24] and moving gratings [25]. In the present study, distractors were not as salient. However, they were salient enough to elicit a slight drop in blink rate, an increase in saccade rate, and pupil dilation (see S1 Fig). Importantly, the pupil dilation was present in multiplication trials but not in passive viewing trials. As pupil dilations are associated with work load increase [26–28], this pupil dilation in reaction to distractors might reflect instantaneous attempts to prevent interference by the distractor.

Maybe only distractors of higher saliency (e.g. moving numbers) are able to break through the desensitization to sensory information (perceptual decoupling) activated by a highly demanding internal task. As desensitization might not be enough to prevent attentional capture by highly salient distractors, one actively looks away. In other words, only when saliency of distractors are beyond a certain threshold, additional active shielding mechanisms (blinking and gaze aversion) may be recruited.

Participants perceived the multiplications with distractor as more challenging and interesting than the multiplications without distraction. The expected appearance of irrelevant distractors might have motivated them to engage stronger in the task, which in turn might further reduce their sensitivity to distractors. Buetti and Lleras [1] propose in their *Engagement Theory of Distractibility* that the level of engagement and performance in a mental task determines the distractibility by irrelevant visual distractors and not vice versa. A similar idea was proposed Humphrey and Revelle in 1984 [42] regarding information transfer tasks (tasks requiring reaction to stimuli).

If participants tried harder, we would have expected changes at the level of pupil diameter and microsaccade rate [26,28,29]. However, neither pupil diameter nor microsaccade rate indicated any differences in workload or arousal between the multiplications with and without distraction.

The *Engagement Theory of Distractibility* [1] may only apply, when the internal task requires little to medium cognitive resources over a prolonged time allowing for variations in the strength of internal focus and therefore variations in perceptual decoupling. In the study of Buetti and Lleras (2016), a three-digit number appeared at the beginning of a trial and every 1.3 to 3.41s an operation (e.g. “plus 5”, “minus 2”, “times 1”) was presented on the screen or auditorily. On average 2.3 s after presentation of a new operation, a new irrelevant picture appeared on the screen. As the hardest operation in Buetti and Lleras (2016) study was multiplying by factor 2, we can assume shorter task performance times than actual inter-stimulus-interval, which leaves more time for shifts in attentional focus to distraction.

If an internal task goes on for a prolonged time, arousal levels could start varying and deviating from optimal levels and making one prone to mind wandering and external distraction [27,28]. In tasks requiring speeded responses to stimuli, increased task engagement (through try-harder instructions) reduces occasional breakdowns of information processing leading to a more stable information-processing rate [42,43]. Similarly, in non-speeded internal tasks, increasing task engagement could stabilize arousal levels, thereby reducing proneness to distraction. This reduced proneness to distraction could lead to higher performance accuracy, as distractors are less able to interfere with internal representations.

In the present study, the internal task of multiplications required a relatively high amount of cognitive resources for a short period (it took participants on average less than 3 s to solve the multiplications). Maybe the distractor presence did not encourage even stronger task engagement simply because the internal task already required such a high level of cognitive resources that perceptual decoupling was at maximum and distractors were therefore effectively gated out.

We initially considered including trials with lower demands (i.e. easier calculations) and ran some pilot tests during planning of the current study. However, pilot-participants were simply faster in calculating easier calculations (ca. 1s), still not noticing the distractors. As pilot-participants reported not noticing the distractors in the easier calculations either, we assume that easier calculations elicit a similarly strong perceptual decoupling as harder calculations, just for a shorter time. The shorter calculation periods required earlier presentation of distractors and therefore smaller analysis windows (time in which both the conditions without and with distractor are identical regarding visual display). We therefore chose the harder multiplications in the present study. Alternatively, future studies could also use tasks that are less demanding and require continuous performance over a longer time like the alternate uses task [44].

Results from perceptual decoupling, gaze aversion and task engagement studies seem to be rather complimentary than competing. The *Perceptual Decoupling hypothesis* [7] describes the desensitization to sensory information irrespective of their relevance as soon as attention turns inward. The *Engagement Theory of Distractibility* [1] may apply when the task allows for variation in arousal and focus (e.g. because of long duration and low to medium workload) and those variations can be stabilized by stronger task engagement. Moreover, active ocular shielding mechanisms (e.g. gaze aversion; [24,25]) may only play a role when saliency of distraction exceeds the threshold of the desensitization by task focus.

### Limitations and future directions

In the present study, all stimuli were presented visually. We were especially interested how eye behavior is associated with shielding the internal train of thought from external (visual) stimulation and whether the eyes play a functional role in that. As eyes are the sensory organ for visual information, it was intuitive for us to focus on visual stimulation (especially for distractors). The short visual presentation of the operands required participants’ focused attention and guaranteed that processing during the EDC time window was challenging and not confounded with task unrelated thought (e.g. mind wandering). At the same time, the visual presentation guaranteed that the eyes showed a pattern of visual information intake. An auditory presentation of the operands would not require the eyes to focus on visual information and would have made it hard to compare EDC to the calculation phase in which visual information is ignored. To avoid a modality shift and to be able to compare a phase of visual information intake to a phase of decoupling from visual input, we used visual stimuli only.

However, further studies should investigate if the decoupling of eye behavior is actually a phenomenon specifically associated with the shifting from EDC to IDC, or if it occurs in the context of shifting attention from external visual information to any form of nonvisual information (internal or external). As different information processing from different sensory modalities typically interfere (e.g. attending to visual and auditory information), we might expect that an attentional focus on external auditory information may also induce decoupling of eye behavior to shield it from visual interference.

As there was no prior research on potential effects of expected distraction, we had no information on whether eyes would adapt at all and if they did how fast such an adaption would occur. It might takes some time (e.g. several trials) until anticipation of distraction is strong enough to have an effect on eye behavior. Therefore, we employed a block design to study both fast and slow (over several trials) adaptions of eye behavior. Further, the block design ensured that participants will quickly learn that distractors actually occur, giving this experimental manipulation high face validity. As a potential disadvantage of a block design, conditions with incongruent warnings (“no distractor” when there actually are distractors and vice versa) do not work, simply because any violation to the announced condition would undermine strong and unambiguous expectations about whether distractors will occur or not. The strength of our design was that by comparing only the first 1.5 s of each calculation period, in which both conditions were identical (no distractor yet), any effects of actual distractor appearance on eye behavior (e.g., effects of luminance…) were avoided.

In the present study, distractors could appear at one of five intervals. Individuals are known to pick up these implicit time-related aspects of the trial structure and to use them to improve their reaction-time performance at following trials [45]. The same probability monitoring process could differentially modulate distractor processing at different delays. However, probability-monitoring processes require cognitive resources. As irrelevant auditory stimulation was shown to bind enough cognitive resources to impair conditional-probability monitoring [45], calculating multiplications should bind even more cognitive resources and impair conditional-probability monitoring even more. Therefore, we do not expect that participants effectively used distractors conditional probability to modulate distractor processing at different delays in this study. Future studies using internal tasks that leave enough cognitive resources for probability monitoring processes, could exploit conditional probability monitoring effects to study expectancy effects parametrically beyond mere presence versus absence of distractors.

### Conclusion

Taken together, the present study showed that decoupling from external visual events can be observed at the level of eye behavior. Importantly, perceptual decoupling is not further modulated by expected distraction, at least for short highly demanding internal tasks. In other words, perceptual decoupling happens in a largely automatic, non-adaptive fashion, independent of the level of expected visual distraction. The automatic perceptual decoupling response observed at the level of eye behavior may be the result of a centrally mediated gating function as proposed by the *Prefrontal-Basal Ganglia Working Memory Model* [15]. Focused IDC was shown to be associated with increased EEG alpha activity [46,47] as well as reduced brain activity at visual cortex mediated by the right inferior parietal cortex [48], which may reflect neural correlates of such a top-down desensitization mechanism leading to reduced cortical processing of sensory stimuli [12].

This general perceptual decoupling has important implications for everyday life. It is helpful in situations in which the main cognitive activity requires internal attention and the surrounding environment is irrelevant, such as when thinking about a good closing sentence for your manuscript while sitting in the park, as you do not have to adapt your perceptual decoupling to the changing number of kids running around. However, sometimes a largely automatic perceptual decoupling may also be problematic or even dangerous. External stimuli may become relevant without changing their visual saliency, for example, when one of the kids’ water balloons flies towards your laptop.

Taken together, the present study suggests that whether distractors are expected or not has no effect on the strength of decoupling from external visual events during IDC. This result further supports the *Perceptual Decoupling hypothesis*: focusing inward automatically induces desensitization to external events independent of the actual external environment.

## Supporting information

S1 Fig Eye behavior in reaction to first distractor. Eye parameters were baseline corrected using the 500ms prior distractor appearance and are plotted relative to distractor appearance. Error bars indicate 95% confidence intervals.
(TIF)

S1 Table Planned comparisons of the general effect of turning attention inward. Descriptive statistic and planned comparisons of the general effect of turning attention inward from reading operands (-0.5s to 0s) to calculating the multiplication in mind for each task separately.
(DOCX)

S2 Table Passive viewing vs. multiplications without and with distractor. Planned comparisons of eye behavior during passive viewing vs. multiplications without and with distractor.
(DOCX)

S3 Table Details on multiplications. Items, correct and false answers.
(DOCX)

S4 Table Average correlations between eye parameters. (DOCX)
