# Supplementary figures and images for: Eye behavior does not adapt to expected visual distraction during internally directed cognition
Source: PLoS One. 2018 Sep 28;13(9):e0204963. doi: 10.1371/journal.pone.0204963 (PMC6161918; doi:10.1371/journal.pone.0204963)

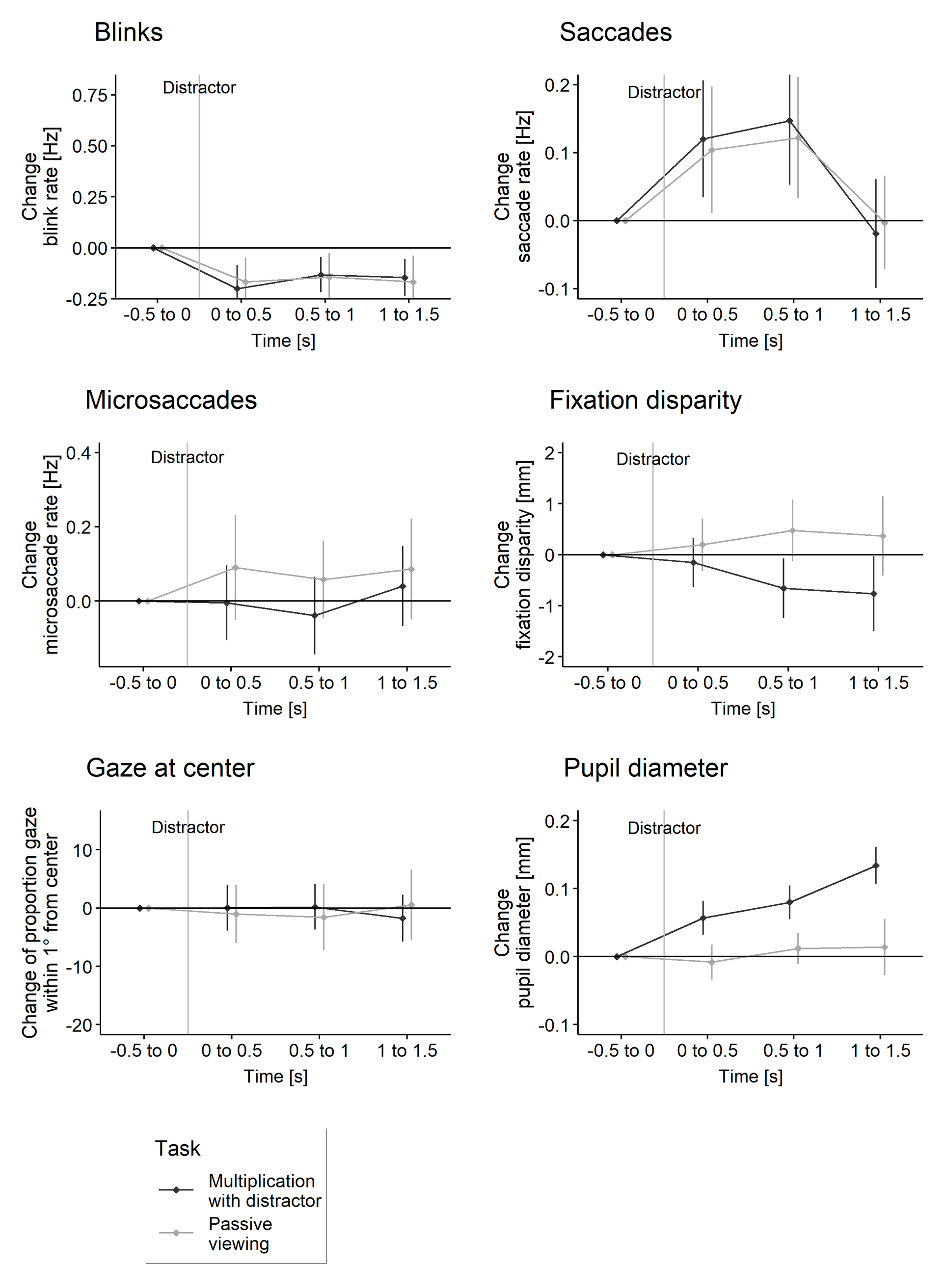

Supplement: S1 Fig — Eye parameters were baseline corrected using the 500ms prior distractor appearance and are plotted relative to distractor appearance. Error bars indicate 95% confidence intervals. (TIF) [file pone.0204963.s001.tif]
