# Supplementary material for: Eye behavior does not adapt to expected visual distraction during internally directed cognition
Source: PLoS One. 2018 Sep 28;13(9):e0204963. doi: 10.1371/journal.pone.0204963 (PMC6161918; doi:10.1371/journal.pone.0204963)
Supplement: S1 Table — Descriptive statistic and planned comparisons of the general effect of turning attention inward from reading operands (-0.5s to 0s) to calculating the multiplication in mind for each task separately. (DOCX) [file pone.0204963.s002.docx]

| **S1 Table. Planned comparisons of the general effect of turning attention inward.** | | | | | | | | | |
| --- | --- | --- | --- | --- | --- | --- | --- | --- | --- |
| Eye parameter | Condition | Time | *M* | *SD* | *t*^a^ | *p*^ab^ | Cohen’s *d*^a^ | *BF*_10_ | *BF*_01_ |
| *Blink rate* | Multiplication without distractor | -0.5 to 0s | -0.04 | 0.27 |  |  |  |  |  |
|  |  | 0s to 0.5s | 0.57 | 0.49 | **5.51** | < .001 | 0.92 | 5490.28 | 0 |
|  |  | 0.5s to 1s | 0.47 | 0.30 | **6.52** | < .001 | 1.09 | 95012.4 | 0 |
|  |  | 1s to 1.5s | 0.34 | 0.31 | **5.74** | < .001 | 0.96 | 10671.63 | 0 |
|  | Multiplication with distractor | -0.5 to 0s | -0.02 | 0.29 |  |  |  |  |  |
|  |  | 0s to 0.5s | 0.62 | 0.52 | **5.56** | < .001 | 0.93 | 6429.06 | 0 |
|  |  | 0.5s to 1s | 0.44 | 0.31 | **6.41** | < .001 | 1.07 | 70017.05 | 0 |
|  |  | 1s to 1.5s | 0.29 | 0.40 | **3.83** | .002 | 0.64 | 57.69 | 0.02 |
|  | Passive viewing | -0.5 to 0s | -0.04 | 0.25 |  |  |  |  |  |
|  |  | 0s to 0.5s | 0.17 | 0.40 | **2.65** | .036 | 0.44 | 3.62 | 0.28 |
|  |  | 0.5s to 1s | 0.03 | 0.26 | 1.90 | .197 | 0.32 | 0.9 | 1.11 |
|  |  | 1s to 1.5s | 0.05 | 0.34 | 1.89 | .202 | 0.31 | 0.88 | 1.14 |
| *Saccade rate* | Multiplication without distractor | -0.5 to 0s | -0.01 | 0.10 |  |  |  |  |  |
|  |  | 0s to 0.5s | 0.03 | 0.08 | 1.61 | .353 | 0.27 | 0.57 | 1.74 |
|  |  | 0.5s to 1s | 0.07 | 0.10 | **2.77** | .026 | 0.46 | 4.71 | 0.21 |
|  |  | 1s to 1.5s | 0.07 | 0.09 | **3.19** | .009 | 0.53 | 11.94 | 0.08 |
|  | Multiplication with distractor | -0.5 to 0s | 0.01 | 0.09 |  |  |  |  |  |
|  |  | 0s to 0.5s | 0.06 | 0.13 | 1.62 | .344 | 0.27 | 0.59 | 1.71 |
|  |  | 0.5s to 1s | 0.12 | 0.17 | **2.96** | .016 | 0.49 | 7.14 | 0.14 |
|  |  | 1s to 1.5s | 0.13 | 0.24 | 2.44 | .059 | 0.41 | 2.38 | 0.42 |
|  | Passive viewing | -0.5 to 0s | 0.02 | 0.14 |  |  |  |  |  |
|  |  | 0s to 0.5s | 0.00 | 0.11 | -0.81 | 1 | -0.14 | 0.24 | 4.11 |
|  |  | 0.5s to 1s | 0.04 | 0.13 | 0.68 | 1 | 0.11 | 0.22 | 4.49 |
|  |  | 1s to 1.5s | 0.01 | 0.11 | -0.64 | 1 | -0.11 | 0.22 | 4.63 |
| *Micro-saccade rate* | Multiplication without distractor | -0.5 to 0s | -0.06 | 0.17 |  |  |  |  |  |
|  |  | 0s to 0.5s | 0.05 | 0.18 | **3.63** | .003 | 0.60 | 34.43 | 0.03 |
|  |  | 0.5s to 1s | 0.20 | 0.25 | **5.08** | < .001 | 0.85 | 1643.41 | 0 |
|  |  | 1s to 1.5s | 0.21 | 0.25 | **5.75** | < .001 | 0.96 | 10876.71 | 0 |
|  | Multiplication with distractor | -0.5 to 0s | -0.05 | 0.19 |  |  |  |  |  |
|  |  | 0s to 0.5s | 0.06 | 0.19 | **3.01** | .014 | 0.50 | 7.92 | 0.13 |
|  |  | 0.5s to 1s | 0.24 | 0.42 | **3.39** | .005 | 0.56 | 19.12 | 0.05 |
|  |  | 1s to 1.5s | 0.23 | 0.30 | **4.96** | < .001 | 0.83 | 1198.77 | 0 |
|  | Passive viewing | -0.5 to 0s | -0.04 | 0.12 |  |  |  |  |  |
|  |  | 0s to 0.5s | 0.01 | 0.17 | 1.79 | .248 | 0.30 | 0.75 | 1.33 |
|  |  | 0.5s to 1s | 0.06 | 0.27 | 2.45 | .058 | 0.41 | 2.42 | 0.41 |
|  |  | 1s to 1.5s | 0.07 | 0.29 | 2.26 | .091 | 0.38 | 1.68 | 0.6 |
| Fixation disparity | Multiplication without distractor | -0.5 to 0s | 0.13 | 1.35 |  |  |  |  |  |
|  |  | 0s to 0.5s | 0.23 | 1.31 | 0.41 | 1 | 0.07 | 0.19 | 5.17 |
|  |  | 0.5s to 1s | 0.69 | 1.55 | 1.57 | .380 | 0.26 | 0.54 | 1.84 |
|  |  | 1s to 1.5s | 0.43 | 1.84 | 0.73 | 1 | 0.12 | 0.23 | 4.38 |
|  | Multiplication with distractor | -0.5 to 0s | 0.02 | 1.49 |  |  |  |  |  |
|  |  | 0s to 0.5s | -0.06 | 1.26 | -0.34 | 1 | -0.06 | 0.19 | 5.29 |
|  |  | 0.5s to 1s | 0.41 | 1.67 | 1.17 | .751 | 0.19 | 0.34 | 2.98 |
|  |  | 1s to 1.5s | 0.00 | 1.82 | -0.08 | 1 | -0.01 | 0.18 | 5.57 |
|  | Passive viewing | -0.5 to 0s | -0.11 | 1.38 |  |  |  |  |  |
|  |  | 0s to 0.5s | 0.13 | 1.45 | 1.01 | .964 | 0.17 | 0.29 | 3.5 |
|  |  | 0.5s to 1s | -0.06 | 1.71 | 0.17 | 1 | 0.03 | 0.18 | 5.51 |
|  |  | 1s to 1.5s | -0.46 | 2.16 | -0.79 | 1 | -0.13 | 0.24 | 4.17 |
| (continued) | | | | | | | | | |

| (continued) | | | | | | | | | | |
| --- | --- | --- | --- | --- | --- | --- | --- | --- | --- | --- |
| Eye parameter | Condition | Time | *M* | *SD* | *t*^a^ | *p*^ab^ | Cohen’s *d*^a^ | *BF*_10_ | *BF*_01_ |  |
| Gaze position | Multiplication without distractor | -0.5 to 0s | 4.24 | 8.85 |  |  |  |  |  |  |
|  |  | 0s to 0.5s | -5.64 | 10.57 | **-5.35** | < .001 | -0.89 | 3499.95 | 0 |  |
|  |  | 0.5s to 1s | -10.69 | 14.08 | **-4.69** | < .001 | -0.78 | 565.01 | 0 |  |
|  |  | 1s to 1.5s | -9.09 | 15.74 | **-3.96** | .001 | -0.66 | 80.49 | 0.01 |  |
|  | Multiplication with distractor | -0.5 to 0s | 3.56 | 9.64 |  |  |  |  |  |  |
|  |  | 0s to 0.5s | -8.15 | 10.14 | **-5.09** | < .001 | -0.85 | 1707.3 | 0 |  |
|  |  | 0.5s to 1s | -14.00 | 14.49 | **-5.30** | < .001 | -0.88 | 3080.46 | 0 |  |
|  |  | 1s to 1.5s | -13.81 | 18.43 | **-4.47** | < .001 | -0.75 | 311.8 | 0 |  |
|  | Passive viewing | -0.5 to 0s | 3.46 | 12.75 |  |  |  |  |  |  |
|  |  | 0s to 0.5s | 0.82 | 12.00 | -1.48 | .445 | -0.25 | 0.48 | 2.07 |  |
|  |  | 0.5s to 1s | -0.15 | 13.52 | -1.63 | .338 | -0.27 | 0.59 | 1.69 |  |
|  |  | 1s to 1.5s | 2.00 | 11.47 | -0.73 | 1 | -0.12 | 0.23 | 4.37 |  |
| Pupil diameter | Multiplication without distractor | -0.5 to 0s | -0.02 | 0.08 |  |  |  |  |  |  |
|  |  | 0s to 0.5s | 0.00 | 0.06 | 1.57 | .375 | 0.26 | 0.55 | 1.82 |  |
|  |  | 0.5s to 1s | 0.10 | 0.08 | **5.97** | < .001 | 1.00 | 20386.59 | 0 |  |
|  |  | 1s to 1.5s | 0.13 | 0.15 | **4.50** | < .001 | 0.75 | 337.74 | 0 |  |
|  | Multiplication with distractor | -0.5 to 0s | -0.02 | 0.10 |  |  |  |  |  |  |
|  |  | 0s to 0.5s | 0.02 | 0.07 | **3.54** | .003 | 0.59 | 27.48 | 0.04 |  |
|  |  | 0.5s to 1s | 0.10 | 0.09 | **5.55** | < .001 | 0.92 | 6135.79 | 0 |  |
|  |  | 1s to 1.5s | 0.15 | 0.14 | **5.25** | < .001 | 0.88 | 2696.36 | 0 |  |
|  | Passive viewing | -0.5 to 0s | -0.03 | 0.09 |  |  |  |  |  |  |
|  |  | 0s to 0.5s | -0.03 | 0.10 | 0.08 | 1 | 0.01 | 0.18 | 5.57 |  |
|  |  | 0.5s to 1s | 0.01 | 0.09 | 2.27 | .088 | 0.38 | 1.72 | 0.58 |  |
|  |  | 1s to 1.5s | 0.00 | 0.12 | 1.31 | .596 | 0.22 | 0.39 | 2.55 |  |
| ^a^ To analyze the effect of turning attention inward to calculate the multiplication, we compared each time bin after operand offset to the time bin during operand presentation (-0.5s to 0s).  ^b^ *p*-values were corrected for 3 tests per eye parameter using Bonferroni-correction.  *df* = 36. Significant *t*-values are highlighted in bold. | | | | | | | | | | |
