# Supplementary material for: Eye behavior does not adapt to expected visual distraction during internally directed cognition
Source: PLoS One. 2018 Sep 28;13(9):e0204963. doi: 10.1371/journal.pone.0204963 (PMC6161918; doi:10.1371/journal.pone.0204963)
Supplement: S2 Table — Planned comparisons of eye behavior during passive viewing vs. multiplications without and with distractor. (DOCX) [file pone.0204963.s003.docx]

| **S2 Table. Passive viewing vs. multiplications without and with distractor.** | | | | | | | |
| --- | --- | --- | --- | --- | --- | --- | --- |
| Eye parameter | Comparison | Time | *t* | *p*^a^ | Cohen’s *d* | *BF*_10_ | *BF*_01_ |
| Blink rate | Passive viewing vs. multiplication without distractor | -0.5 to 0s | 0.01 | 1 | 0 | 0.18 | 5.59 |
|  |  | 0s to 0.5s | -4.00 | .001 | -.67 | 89.36 | 0.01 |
|  |  | 0.5s to 1s | -5.64 | < .001 | -.94 | 7842.72 | 0 |
|  |  | 1s to 1.5s | -4.56 | < .001 | -.76 | 396.1 | 0 |
|  | Passive viewing vs. multiplication with distractor | -0.5 to 0s | -0.63 | 1 | -.11 | 0.22 | 4.63 |
|  |  | 0s to 0.5s | -4.33 | < .001 | -.72 | 215.32 | 0 |
|  |  | 0.5s to 1s | -5.74 | < .001 | -.96 | 10400.29 | 0 |
|  |  | 1s to 1.5s | -2.86 | .028 | -.48 | 5.68 | 0.18 |
| Saccade rate | Passive viewing vs. multiplication without distractor | -0.5 to 0s | 1.47 | .600 | .25 | 0.48 | 2.08 |
|  |  | 0s to 0.5s | -1.04 | 1 | -.17 | 0.3 | 3.38 |
|  |  | 0.5s to 1s | -0.98 | 1 | -.16 | 0.28 | 3.57 |
|  |  | 1s to 1.5s | -2.44 | .080 | -.41 | 2.37 | 0.42 |
|  | Passive viewing vs. multiplication with distractor | -0.5 to 0s | 0.62 | 1 | .10 | 0.21 | 4.67 |
|  |  | 0s to 0.5s | -1.55 | .521 | -.26 | 0.53 | 1.88 |
|  |  | 0.5s to 1s | -2.00 | .212 | -.33 | 1.06 | 0.94 |
|  |  | 1s to 1.5s | -2.36 | .095 | -.39 | 2.04 | 0.49 |
| Microsaccade rate | Passive viewing vs. multiplication without distractor | -0.5 to 0s | 0.94 | 1 | .16 | 0.27 | 3.72 |
|  |  | 0s to 0.5s | -1.03 | 1 | -.17 | 0.29 | 3.42 |
|  |  | 0.5s to 1s | -2.04 | .198 | -.34 | 1.12 | 0.89 |
|  |  | 1s to 1.5s | -2.12 | .166 | -.35 | 1.3 | 0.77 |
|  | Passive viewing vs. multiplication with distractor | -0.5 to 0s | 0.48 | 1 | .08 | 0.2 | 5.02 |
|  |  | 0s to 0.5s | -1.07 | 1 | -.18 | 0.3 | 3.28 |
|  |  | 0.5s to 1s | -1.96 | .231 | -.33 | 0.99 | 1.01 |
|  |  | 1s to 1.5s | -2.24 | .126 | -.37 | 1.62 | 0.62 |
| Fixation disparity | Passive viewing vs. multiplication without distractor | -0.5 to 0s | -0.84 | 1 | -.14 | 0.25 | 4.01 |
|  |  | 0s to 0.5s | -0.31 | 1 | -.05 | 0.19 | 5.34 |
|  |  | 0.5s to 1s | -1.72 | .375 | -.29 | 0.68 | 1.47 |
|  |  | 1s to 1.5s | -1.87 | .281 | -.31 | 0.85 | 1.17 |
|  | Passive viewing vs. multiplication with distractor | -0.5 to 0s | -0.46 | 1 | -.08 | 0.2 | 5.06 |
|  |  | 0s to 0.5s | 0.56 | 1 | .09 | 0.21 | 4.84 |
|  |  | 0.5s to 1s | -1.00 | 1 | -.17 | 0.28 | 3.53 |
|  |  | 1s to 1.5s | -0.9 | 1 | -.15 | 0.26 | 3.85 |
| Gaze position | Passive viewing vs. multiplication without distractor | -0.5 to 0s | -0.43 | 1 | -.07 | 0.19 | 5.13 |
|  |  | 0s to 0.5s | 2.48 | .072 | .41 | 2.57 | 0.39 |
|  |  | 0.5s to 1s | 2.82 | .031 | .47 | 5.23 | 0.19 |
|  |  | 1s to 1.5s | 2.92 | .024 | .49 | 6.47 | 0.15 |
|  | Passive viewing vs. multiplication with distractor | -0.5 to 0s | -0.06 | 1 | -.01 | 0.18 | 5.58 |
|  |  | 0s to 0.5s | 3.51 | .005 | .59 | 25.82 | 0.04 |
|  |  | 0.5s to 1s | 3.48 | .005 | .58 | 23.93 | 0.04 |
|  |  | 1s to 1.5s | 3.64 | .004 | .61 | 35.16 | 0.03 |
| Pupil diameter | Passive viewing vs. multiplication without distractor | -0.5 to 0s | -0.72 | 1 | -.12 | 0.23 | 4.4 |
|  |  | 0s to 0.5s | -1.74 | .366 | -.29 | 0.69 | 1.44 |
|  |  | 0.5s to 1s | -4.05 | .001 | -.68 | 102.01 | 0.01 |
|  |  | 1s to 1.5s | -3.59 | .004 | -.60 | 31.06 | 0.03 |
|  | Passive viewing vs. multiplication with distractor | -0.5 to 0s | -0.35 | 1 | -.06 | 0.19 | 5.28 |
|  |  | 0s to 0.5s | -1.78 | .335 | -.30 | 0.74 | 1.35 |
|  |  | 0.5s to 1s | -3.41 | .007 | -.57 | 20.09 | 0.05 |
|  |  | 1s to 1.5s | -4.41 | < .001 | -.73 | 260.89 | 0 |
| Planned comparisons of eye behavior during passive viewing vs. multiplications without and with distractor. | | | | | | | |
| a p-values were corrected for 4 tests per eye parameter using Bonferroni-correction. | | | | | | | |
| df = 35. Significant t-values are highlighted in bold. | | | | | | | |
