# Supplementary material for: Eye behavior does not adapt to expected visual distraction during internally directed cognition
Source: PLoS One. 2018 Sep 28;13(9):e0204963. doi: 10.1371/journal.pone.0204963 (PMC6161918; doi:10.1371/journal.pone.0204963)
Supplement: S3 Table — Items, correct and false answers. (DOCX) [file pone.0204963.s004.docx]

| **S3 Table. Details on multiplications.** | | | | | | | |
| --- | --- | --- | --- | --- | --- | --- | --- |
| Set | Task | Trial ID | First operand | Second operand | Correct answer | False answers | Carry-over |
| Practice | multiplication | -3 | 13 | 8 | 104 | [181,189,140,178,198,410,148] | yes |
| Practice | multiplication | -2 | 14 | 7 | 98 | [92,94,96,99,90,89,88] | yes |
| Practice | passive viewing | -1 | 17 | 5 | 85 | [80,81,82,83,86,88,95] | yes |
| Set 1 | multiplication | 1 | 18 | 7 | 126 | [121,162,180,136,198,106,148] | yes |
| Set 1 | multiplication | 2 | 24 | 8 | 192 | [221,162,280,336,198,106,148] | yes |
| Set 1 | multiplication | 3 | 27 | 7 | 189 | [270,248,241,217,277,223,234] | yes |
| Set 1 | multiplication | 4 | 29 | 4 | 116 | [171,93,124,175,119,167,102] | yes |
| Set 1 | multiplication | 5 | 32 | 8 | 256 | [289,283,345,486,171,273,391] | yes |
| Set 1 | multiplication | 6 | 35 | 6 | 210 | [382,283,385,486,171,273,391] | yes |
| Set 1 | multiplication | 7 | 36 | 7 | 252 | [182,283,385,186,171,473,191] | yes |
| Set 1 | multiplication | 8 | 45 | 7 | 315 | [322,423,312,351,314,423,319] | yes |
| Set 1 | multiplication | 9 | 48 | 6 | 288 | [182,283,385,486,171,273,391] | yes |
| Set 1 | multiplication | 10 | 58 | 7 | 406 | [488,443,385,486,471,273,391] | yes |
| Set 1 | multiplication | 11 | 59 | 6 | 354 | [270,208,209,217,277,223,234] | yes |
| Set 1 | multiplication | 12 | 69 | 3 | 207 | [270,208,209,217,277,223,234] | yes |
| Set 1 | multiplication | 13 | 69 | 4 | 276 | [481,189,180,478,188,942,294] | yes |
| Set 1 | multiplication | 14 | 78 | 3 | 234 | [383,287,380,278,188,942,294] | yes |
| Set 1 | multiplication | 15 | 83 | 6 | 498 | [482,283,185,486,271,273,391] | yes |
| Set 1 | multiplication | 16 | 88 | 6 | 528 | [582,483,385,281,111,463,491] | yes |
| Set 1 | multiplication | 17 | 97 | 5 | 485 | [122,423,312,651,514,423,319] | yes |
| Set 1 | multiplication | 18 | 12 | 9 | 108 | [101,189,180,178,198,106,148] | yes |
| Set 1 | passive viewing | 19 | 38 | 6 | 228 | [226,218,238,224,212,198,196] | yes |
| Set 1 | passive viewing | 20 | 79 | 5 | 395 | [385,375,365,295,285,265,355] | yes |
| Set 1 | passive viewing | 21 | 54 | 7 | 378 | [368,358,388,278,268,298,397] | yes |
| Set 1 | passive viewing | 22 | 21 | 9 | 189 | [188,179,168,169,129,139,149] | no |
| Set 2 | multiplication | 23 | 15 | 6 | 90 | [95,99,96,79,77,87,71] | yes |
| Set 2 | multiplication | 24 | 16 | 6 | 96 | [95,99,69,79,77,87,71] | yes |
| Set 2 | multiplication | 25 | 21 | 8 | 168 | [186,189,180,178,198,242,294] | no |
| Set 2 | multiplication | 26 | 27 | 3 | 81 | [12,15,19,27,71,72,16] | yes |
| Set 2 | multiplication | 27 | 27 | 9 | 243 | [281,389,270,478,188,942,294] | yes |
| Set 2 | multiplication | 28 | 33 | 6 | 198 | [181,189,180,178,188,942,294] | yes |
| Set 2 | multiplication | 29 | 33 | 9 | 297 | [270,279,209,217,277,223,234] | yes |
| Set 2 | multiplication | 30 | 36 | 9 | 324 | [322,423,342,344,314,423,339] | yes |
| Set 2 | multiplication | 31 | 42 | 7 | 294 | [481,189,180,478,188,942,194] | yes |
| Set 2 | multiplication | 32 | 43 | 6 | 258 | [381,339,280,478,188,543,204] | yes |
| Set 2 | multiplication | 33 | 49 | 8 | 392 | [382,383,185,286,371,173,491] | yes |
| Set 2 | multiplication | 34 | 49 | 5 | 245 | [244,208,209,217,254,223,234] | yes |
| Set 2 | multiplication | 35 | 66 | 6 | 396 | [481,189,180,478,188,842,324] | yes |
| Set 2 | multiplication | 36 | 68 | 3 | 204 | [296,289,380,478,188,942,294] | yes |
| Set 2 | multiplication | 37 | 76 | 6 | 456 | [322,423,312,351,514,423,319] | yes |
| Set 2 | multiplication | 38 | 76 | 4 | 304 | [382,183,285,486,171,173,191] | yes |
| Set 2 | multiplication | 39 | 94 | 3 | 282 | [181,189,180,178,198,942,294] | yes |
| Set 2 | multiplication | 40 | 96 | 4 | 384 | [188,433,285,386,721,673,291] | yes |
| Set 2 | passive viewing | 41 | 61 | 6 | 366 | [356,346,336,266,256,376,286] | no |
| Set 2 | passive viewing | 42 | 72 | 9 | 648 | [658,638,668,548,558,518,638] | yes |
| Set 2 | passive viewing | 43 | 34 | 6 | 204 | [214,194,184,224,242,240,206] | yes |
| Set 2 | passive viewing | 44 | 86 | 3 | 258 | [248,268,278,238,158,168,155] | yes |
| Items were generated and then randomly assigned to multiplication and passive viewing conditions and to set 1 and set 2. One easier multiplication item with and one without carry-over as well as a passive viewing item were generated for practice trial. Order of sets and assignment to condition (with or without distractor) were counterbalanced across participants. Order of trials within a set was randomized for each participant. | | | | | | | |
