# Supplementary material for: Eye behavior does not adapt to expected visual distraction during internally directed cognition
Source: PLoS One. 2018 Sep 28;13(9):e0204963. doi: 10.1371/journal.pone.0204963 (PMC6161918; doi:10.1371/journal.pone.0204963)
Supplement: S4 Table — (DOCX) [file pone.0204963.s005.docx]

| **S4 Table. Average correlations between eye parameters.** | | | | | |
| --- | --- | --- | --- | --- | --- |
| Variable | Pupil diameter | Fixation disparity | Saccade rate | Blink rate | Microsaccade rate |
|  |  |  |  |  |  |
| Pupil diameter |  |  |  |  |  |
|  |  |  |  |  |  |
| Fixation disparity | -.11 |  |  |  |  |
|  | [-.72, .26] |  |  |  |  |
|  |  |  |  |  |  |
| Saccade rate | .04 | .02 |  |  |  |
|  | [-.17, .29] | [-.18, .25] |  |  |  |
|  |  |  |  |  |  |
| Blink rate | .00 | .01 | .06 |  |  |
|  | [-.31, .27] | [-.20, .23] | [-.10, .43] |  |  |
|  |  |  |  |  |  |
| Microsaccade rate | .05 | .04 | .07 | .10 |  |
|  | [-.18, .24] | [-.17, .28] | [-.12, .54] | [-.15, .52] |  |
|  |  |  |  |  |  |
| Gaze position | .02 | -.08 | -.17 | -.42 | -.12 |
|  | [-.50, .43] | [-.36, .23] | [-.49, .11] | [-.84, -.07] | [-.38, .14] |
|  |  |  |  |  |  |
| Values represent mean correlation coefficient. Minimum and maximum correlation coefficients are presented in square brackets. Correlation coefficients reflect average within-subject correlation of eye parameters. | | | | | |
